# A disease-specific measure of health-related quality of life for use in adults with immune thrombocytopenic purpura: Its development and validation

**DOI:** 10.1186/1477-7525-5-11

**Published:** 2007-02-22

**Authors:** Susan D Mathias, James B Bussel, James N George, Robert McMillan, Gary J Okano, Janet L Nichol

**Affiliations:** 1 Ovation Research Group, 188 Embarcadero, Suite 200, San Francisco, CA 94105, USA; 2 New York Presbyterian Hospital/Weill Cornell Medical Center, 525 East 68th Street, New York, NY 10021, USA; 3 University of Oklahoma Health Sciences Center, P.O. Box 26901, Oklahoma City, OK 73190, USA; 4 The Scripps Research Institute, 10550 N Torrey Pines Road, La Jolla, CA 92037, USA; 5 Amgen, Inc. One Amgen Center Drive, MS: 28-3-A, Thousand Oaks, CA 91320-1799, USA

## Abstract

**Background:**

No validated disease-specific measures are available to assess health-related quality of life (HRQoL) in adult subjects with immune thrombocytopenic purpura (ITP). Therefore, we sought to develop and validate the ITP-Patient Assessment Questionnaire (ITP-PAQ) for adult subjects with ITP.

**Methods:**

Information from literature reviews, focus groups with subjects, and clinicians were used to develop 50 ITP-PAQ items. Factor analyses were conducted to develop the scale structure and reduce the number of items. The final 44-item ITP-PAQ, which includes ten scales [Symptoms (S), Bother-Physical Health (B), Fatigue/Sleep (FT), Activity (A), Fear (FR), Psychological Health (PH), Work (W), Social Activity (SA), Women's Reproductive Health (RH), and Overall (QoL)], was self-administered to adult ITP subjects at baseline and 7–10 days later. Test-retest reliability, internal consistency reliability, construct and known groups validity of the final ITP-PAQ were evaluated.

**Results:**

Seventy-three subjects with ITP completed the questionnaire twice. Test-retest reliability, as measured by the intra-class correlation, ranged from 0.52–0.90. Internal consistency reliability was demonstrated with Cronbach's alpha for all scales above the acceptable level of 0.70 (range: 0.71–0.92), except for RH (0.66). Construct validity, assessed by correlating ITP-PAQ scales with established measures (Short Form-36 v.1, SF-36 and Center for Epidemiologic Studies Depression Scale, CES-D), was demonstrated through moderate correlations between the ITP-PAQ SA and SF-36 Social Function scales (r = 0.67), and between ITP-PAQ PH and SF-36 Mental Health Scales (r = 0.63). Moderate to strong inter-scale correlations were reported between ITP-PAQ scales and the CES-D, except for the RH scale. Known groups validity was evaluated by comparing mean scores for groups that differed clinically. Statistically significant differences (p < 0.01) were observed when subjects were categorized by treatment status [S, FT, B, A, PH, and QoL, perceived effectiveness of ITP treatment [S], and time elapsed since ITP diagnosis [PH].

**Conclusion:**

Results provide preliminary evidence of the reliability and validity of the ITP-PAQ in adult subjects with ITP. Further work should be conducted to assess the responsiveness and to estimate the minimal clinical important difference of the ITP-PAQ to more fully understand the impact of ITP and its treatments on HRQoL.

## Background

Immune thrombocytopenic purpura (ITP) is a disorder characterized by autoimmune-mediated platelet destruction and suboptimal platelet production [1-3] that results in a decrease in the number of circulating platelets and increases the risk of bleeding events. The estimated prevalence rate for ITP in the United States is 9.5/100,000 [4]. Adult women are disproportionately affected by the disorder, with a female to male ratio of nearly two to one [5]. The disorder rarely remits spontaneously in adult subjects [1]. The mortality rate is relatively low (< 1%) [6] in adults less than 65 years of age. Morbidity increases above age 65, primarily as a result of an increase in age-related major bleeding events [7].

Initial therapy for ITP consists of some combination of glucocorticoids, intravenous immune globulin (IVIg) or anti-D [8]. Splenectomy is often considered if these therapies fail. Approximately two-thirds of patients treated with splenectomy achieve a sustained remission [1,5,9]. Patients who fail splenectomy are treated with a wide variety of agents including corticosteroids, danazol, and chemotherapeutic agents. Morbidity and mortality in these refractory patients are substantial [1,6,8].

Patient-reported outcomes (PRO), including health-related quality of life (HRQoL) measures, are critical components for evaluating and understanding treatment effects from the subject's perspective. The Food and Drug Administration (FDA) indicates that PRO measures are important to assess because they may: 1) detect treatment effects known only to the subject; 2) understand the subject's perspective regarding treatment effect; or 3) provide information not included in a clinician's subject notes [10]. Furthermore, the Committee for Medicinal Products for Human Use of the European Medicines Agency defines HRQoL as "the subject's subjective perception of the impact of his disease and its treatment(s) on his daily life, physical, psychological and social functioning and well-being" [11].

Currently, limited data are available on the assessment of the impact of symptoms in adult subjects with ITP. Symptoms of ITP, such as spontaneous bruising, menorrhagia, mucosal bleeding and prolonged bleeding with injury, may significantly affect HRQoL in ITP subjects [12]. Treatments for chronic ITP can also be associated with substantial side effects [5,8]. In addition, subjects who are resistant to current therapies are likely to experience an even greater decrement to their HRQoL than responders to treatment. Thus, restoring and/or maintaining quality of life should be an important goal of treatment. While the primary markers for ITP include hematologic measures such as platelet counts, clinical measures typically do not assess a subject's functioning and well-being. Therefore, subjects and physicians may want to weigh the impact of ITP therapies on HRQoL endpoints when making treatment decisions.

Previously, no validated disease-specific measures were available to evaluate quality of life in adult ITP subjects; however, two disease-specific HRQoL questionnaires have been developed for use in children with ITP [13,14]. Thus, we sought to develop a questionnaire that would be appropriate to assess the impact of ITP symptoms and its treatments on HRQoL in adult subjects. The objective of this current manuscript is to describe the development and initial validation of a newly developed HRQoL questionnaire for use in adult subjects with ITP.

## Methods

### Overview

A newly developed questionnaire, which assesses issues of importance to ITP subjects, was developed based on available published literature, existing questionnaires, expert clinical opinion, and input from subjects with ITP.

### Subjects and Procedures

To develop the questionnaire, three focus groups with ITP subjects were conducted in geographically diverse locations (San Diego, CA, New York, NY, and Oklahoma City, OK). Each of the three sites recruited a convenience sample of five to eight ITP subjects who were being treated on an outpatient basis. To be eligible, subjects were required to have active disease and be ≥ 18 years of age. Although platelet count data were not required for participation in the focus groups, clinicians at each site considered the subjects to have active disease, usually an indication that platelet levels have dropped below 120 × 10^9^L and the subject requires treatment and/or more frequent monitoring. In total, 23 ITP subjects participated in the focus groups after providing their informed consent.

To validate the questionnaire, a convenience sample of subjects was recruited from the same three clinical sites in New York, NY, Oklahoma City, OK, and La Jolla, CA. Subjects were eligible if they were ≥ 18 years of age, had active disease, and were willing to complete a self-administered questionnaire at two time points. Target enrollment was roughly 72 subjects (24 subjects from each site).

The study protocol was approved by a local institutional review board at each site and was carried out in accordance with Good Clinical Practice and International Conference on Harmonization guidelines and the Declaration of Helsinki. Written informed consent was obtained from each subject prior to enrollment.

### Creation of Questionnaire/Item Selection

A trained moderator led all focus groups using a detailed discussion guide. Subjects discussed their ITP history, treatment, ITP symptoms, and the impact of ITP on daily activities. Each focus group lasted approximately three hours, and subjects were provided with an honorarium for their time. Following the focus group session, all subjects completed a questionnaire which included the SF-36 v1 [15] and ITP-specific questions. The ITP-specific items were developed based on clinical input [12,16-18]. The ITP-specific items assessed the impact of ITP on the subject's overall quality of life, relationships, ability to sleep, menstruation/gynecological history, and sexual activity. The ITP-specific questions also assessed the subject's response to ITP treatments and any side effects. Transcripts of the focus groups were summarized and reviewed by the study team. The initial draft of the ITP Subject Assessment Questionnaire (ITP-PAQ) was developed after reviewing information from the literature searches, existing questionnaires, expert opinion, focus group transcripts, and the questionnaire responses from the focus group subjects.

The initial ITP-PAQ consisted of 50 items that assesses the impact of ITP in the areas of physical health, mental health, work, social activity, reproductive health (relevant for women only), and overall quality of life. Factor analyses were conducted which yielded six unique domains. The impact of ITP on physical health was measured by four scales that evaluated ITP-related symptoms, Fatigue/Sleep, Bother-Physical Health, and Activity. Its impact on mental health was measured by two scales that evaluated psychological distress and fear. A copy of the questionnaire can be obtained by contacting Janet L. Nichol and sample items are included Table 2.

**Table 2:** Overview of ITP-PAQ Scales and Item Variability

**ITP-PAQ Scales**	**No. of Items**	**Mean**	**Range**	**Example Item**
Symptoms	6	3.79 (0.72)	1.86 – 5.00	In the past 4 weeks, how often did you have bleeding episodes (nose bleeds, gum bleeds, etc)?
Bother-Physical Health	3	3.62 (1.01)	1.00 – 5.00	Overall, in the past 4 weeks, how bothered have you been by the effect of ITP and its treatment(s) on your physical health?
Fatigue/Sleep	4	3044 (1.15)	1.25 – 5.00	In the past 4 weeks, how often did ITP or its treatments cause you to feel physically fatigued?
Activity	2	3.55 (1.37)	1.00 – 5.00	In the past 4 weeks, how much has your ITP symptoms or the effects of its treatments interfered with your ability to exercise?
Fear	5	4.22 (0.74)	1.50 – 5.00	In the past 4 weeks, how fearful have you been of having a bleeding episode (nose bleeds, gum bleeds, etc)?
Psychological Health	5	3.59 (0.98)	1.00 – 5.00	In the past 4 weeks, how often did you have feelings of sadness or depression because of your ITP or its treatments?
Work	4	4.13 (0.99)	1.75–5.00	Since you were diagnosed, to what degree has ITP negatively interfered with your choice of career(s)?
Social Activity	4	4.26 (0.65)	2.33 – 5.00	In the past 4 weeks, how often has ITP limited your ability to participate in social activities?
Women's Reproductive Health	6	3.78 (0.98)	1.00 – 5.00	Thinking about your last period, how bothered were you by: heavier bleeding than before having ITP?
Overall QoL	5	3.08 (1.13)	1.00 – 5.00	Overall, in the past 4 weeks, to what extent has ITP and its treatment(s) affected your quality of life?

**Total**	**44**			

### Statistical Analyses

A validation study was conducted to evaluate the psychometric properties of the newly developed ITP-PAQ questionnaire so that it could be used to measure the impact of ITP in adult subjects in future studies. Standard psychometric methods were used to evaluate the reliability and validity of the questionnaire [19,20].

Eligible subjects completed the baseline questionnaire at the site or by mail after providing telephone consent. An informed consent form and baseline questionnaire was mailed to those subjects who gave their initial consent via telephone. These completed documents were returned by mail to the investigators. At follow up, each subject was mailed the same questionnaire and asked to complete it a second time (for evaluating test-retest reliability) approximately two weeks later. Additionally, subjects completed the SF-36 and the CES-D[21] for validation purposes at both assessments. Demographic and clinical characteristics were also solicited in order to more fully describe the study population. Each study subject received an honorarium for completing the questionnaires.

### Scale creation and confirmatory factor analysis

Confirmatory factor analyses were conducted to test the hypothesized structure of the scales. Two models using LISREL version 8 were tested [22]. The first model consisted of all 50 HRQoL items and 10 factors, whereas the second model consisted of a subset of the 50-item correlation matrix. Only women respond to the six items comprising the Reproductive Health scale, so the items were not included in the second LISREL model to avoid estimation biases. The remaining 44 items were analyzed. Model fit was evaluated using the goodness-of-fit (GFI), the normed fit index (NFI), the non-normed fit index (NNFI), the comparative fit index (CIF), and the root mean square error of approximation (RMSEA). For the confirmatory models, index values greater than 0.95 indicate better fit, and RMSEA values less than 0.05 are considered evidence of adequate fit [23].

### Reliability and stability

Two forms of reliability were assessed: test-retest reliability and internal consistency reliability. Test-retest reliability, a measure of the degree to which the questionnaire yields stable scores over a short period of time (assuming there is no underlying change), was measured by the intra-class correlation coefficient (ICC) [24,25]. An ICC of ≥ 0.70 was considered acceptable [26].

Internal consistency reliability, the extent to which items within each scale correlate with each other to form a multi-item scale, was assessed using Cronbach's alpha [25,27]. Data from both assessments were used to evaluate internal consistency reliability. An alpha coefficient of ≥ 0.70 was considered acceptable, which is the commonly accepted minimal standard for reliability coefficients endorsed by the Scientific Advisory Committee of the Medical Outcomes Trust [26].

### Construct Validity

Construct validity was assessed by examining the inter-scale correlations between the ITP-PAQ and the CES-D and the ITP-PAQ with the SF-36 and by examining the strength of the within ITP-PAQ scale correlations [25,28]. For both inter-scale and intra-scale correlations, we made *a priori *hypotheses about the directionality and magnitude of the correlation and observed the extent to which hypothesized relationships held. For example, we hypothesized that the scales of the ITP-PAQ would be negatively correlated with the CES-D and positively correlated with those of the SF-36. We expected the Pearson correlations to be moderate in size.

### Known Groups

Known groups validity evaluates the ability of the measure to discriminate between groups known to be clinically different [28]. We only collected patient-reported information using the questionnaire and did not collect clinical information such as platelet counts. Therefore, the following four criteria were identified as proxies for severity:

• Currently on treatment

• Splenectomy status

• Subjects' self-perception of the effectiveness of current medication

• Length of time since diagnosis

It was hypothesized that subjects not being treated, who did not have a splenectomy, who perceived their medication to be more effective, and who had been diagnosed with ITP for a longer time would be healthier and therefore report higher HRQoL scores. In contrast, subjects on any treatment, who had received a splenectomy, who perceived their medication to be less effective, and who were diagnosed more recently would report worse HRQoL. In addition, subjects were also categorized by gender. Subjects were categorized into two groups for each of the analyses: female vs. male, intact spleen vs. removed spleen, currently on ITP treatment vs. not currently on ITP treatment, subject's perception of the effectiveness of their current ITP medication (extremely/moderately effective vs. not at all effective), and ITP diagnosis less than one year ago vs. ITP diagnosis more than one year ago.

## Results

### Demographics and clinical characteristics

Table 1 describes the demographic and clinical characteristics of the 73 subjects included in the validation analyses. The majority were female (77%) and Caucasian (84%). The mean age was 45 years (SD = 15.7), and most of the subjects had been diagnosed with ITP for at least five years (57%). Fifty-two percent of the subjects reported that they were currently taking medications for their ITP. Furthermore, 58% indicated that they had a splenectomy. Among the 42 subjects who had a splenectomy, 55% reported that the removal of their spleen did not cure their ITP. With one exception, the remaining subjects did not provide a response.

**Table 1:** Demographic and Clinical Characteristics

**Characteristic**	**N = 73**
**Mean Age**	45 years (range, 18–82 years)
**Gender**	
Female	56 (77%)
Male	17 (23%)
**Ethnicity/Race**	
White or Caucasian	61 (84%)
Asian	5 (7%)
Hispanic or Latino	4 (6%)
Black or African American	2 (3%)
American Indian or Alaska Native	1 (1%)
**Marital Status**	
Single, never married	13 (18%)
Married	47 (64 %)
Living with domestic partner/significant other	0 (0%)
Separated	1 (1%)
Divorced	10 (14%)
Widowed	2 (3%)
**Education**	
Less than high school	1 (1%)
High school graduate	13 (18%)
Associate/Technical Degree	13 (18%)
Some college	10 (14%)
Bachelor's degree	18 (25%)
Postgraduate or professional degree	17 (23%)
**Medical Insurance**	
Private insurance	16 (22%)
HMO or PPO	38 (52%)
Medicaid or Medicare	11 (15%)
Other	4 (6%)
No insurance	2 (3%)
**Splenectomy (i.e., spleen removed)**	42 (58%)
**Currently taking medication for ITP**	38 (52%)
**Ever been transfused with blood or platelets for treatment of ITP**	37 (52%)
**Years since initial Diagnosis of ITP**	
< 1 year	13 (18%)
1–2 years	10 (14%)
3–4 years	9 (12%)
5–10 years	15 (21%)
> 10 years	26 (36%)

### Confirmatory factor analysis

The first confirmatory factor analysis of the 50-item and ten factors model converged in 28 iterations. However, neither the inter-item correlation matrix nor the inter-factor correlation matrix was positive-definite, which suggests that the proposed model is wrong for the data or the data are inadequate for the model[22]. The chi-square value of the model was 316.64 with 1129 degrees of freedom (p = 1.0), which does not support the hypothesized scale structure of the initial ITP-PAQ. The confirmatory analysis of this LISREL model indicate that computing domain scores for the Physical Health and Mental Health domains is not appropriate for the ITP-PAQ.

The second LISREL model was analyzed to confirm the scale structure, excluding the Reproductive Health scale. For this model, 126 parameters were estimated: 46 factor loadings, 44 error terms, and 36 inter-factor correlations. The model converged in 39 iterations, with a chi-square value of 1043.10 with 864 degrees of freedom (p < 0.01). The Goodness of Fit Index (GFI), Normed Fit Index (NFI), Non-normed Fit Index (NNFI), and Comparative Fit Index (CFI) was 0.60, 0.63, 0.91, and 0.92, respectively, and the RMSEA was 0.05 [90% CI, 0.04–0.065]. Furthermore, the inter-factor correlations ranged form 0.33 between the Symptoms and Work scales to 0.96 between the Bother-Physical Health and Overall QoL scales.

In addition to the confirmatory factor analyses, Cronbach's alphas and item-to-total correlations were used for item reduction. Items with low factor loadings and item-to-total correlations that reduced the internal consistency were eliminated. Although initial factor analyses identified six domains for future use, the final version of the ITP-PAQ contained 44 items that included the following ten scales: Symptoms, Bother-Physical Health, Fatigue/Sleep, Activity, Fear, Psychological Health, Work, Social Activity, Women's Reproductive Health, and Overall QoL. Table 2 provides information on the number of items, item variability and sample items from each scale of the questionnaire. Each scale is scored from 0 to 100, with higher scores representing better quality of life.

### Test-retest reliability

Of the 73 subjects who completed the first administration of the questionnaire, most of the subjects completed the second questionnaire within a 15-day period (75%), during which subjects were expected to remain clinically stable. However, 20% of the 73 subjects completed the questionnaire within three weeks following the first administration. The remaining 5% of subjects completed it between four and nine weeks after the first "test." ICC's were computed for the entire sample (n = 73) and for a sub-sample of respondents who completed the second questionnaire within three weeks (n = 69). With the exception of the Bother-Physical Health and Activity scales, all scales had acceptable test-retest reliability (ICC ≥ 0.70) as measured by the ICC (Table 3). For the entire sample, ICC values ranged from 0.52–0.90, while ICC values for the sub-sample ranged from 0.56–0.89.

**Table 3:** Test-Retest Reliability (ICCs) and Internal Consistency Reliability (Cronbach's alpha)

**Scales**	ICCs (n = 73)*	**ICCs (n = 69)**^†^	**Cronbach's alpha**
Symptoms	0.80	0.83	0.71
Fatigue/Sleep	0.87	0.88	0.89
Bother-Physical Health	0.52	0.56	0.79
Activity	0.66	0.66	0.89
Psychological	0.72	0.75	0.92
Fear	0.83	0.87	0.87
Social Activity	0.70	0.74	0.72
Work	0.87	0.87	0.86
Reproductive Health	0.90	0.89	0.66
Overall QoL	0.77	0.81	0.89

*Entire sample

^†^Sub-sample of respondents who completed the second questionnaire within three weeks

### Internal consistency reliability

Internal consistency reliability, measured by Cronbach's alpha, ranged from 0.66 to 0.92 (Table 3). With the exception of the Reproductive Health scale, Cronbach's alpha coefficients exceeded the acceptable level of 0.70. Cronbach's alpha for the Symptoms, Bother-Physical Health, Fatigue/Sleep, and Activity scales ranged from 0.71–0.89, while Cronbach's alpha for the Psychological Health and Fear scales ranged from 0.87–0.92. Additionally, Cronbach's alphas for the Social Activity, Work, Reproductive Health, and Overall QoL scales were 0.72, 0.86, 0.66, and 0.89, respectively.

### Construct validity

Table 4 displays the results of inter-scale Pearson correlation coefficients for the initial test administration of the ITP-PAQ. As expected, the Symptoms, Bother-Physical Health, Fatigue/Sleep, and Activity scales were moderately to strongly inter-correlated based on the data from the initial administration (correlation coefficients ranged from 0.56–0.75; p < 0.05). The Overall QoL scale was moderately to strongly correlated with the other ITP-PAQ scales, with the exception of the Reproductive Health scale.

**Table 4:** Construct Validity as measured by Interscale Pearson Correlations*

**Scales**	**S**	**FT**	**B**	**A**	**PH**	**FR**	**SA**	**W**	**RH**	**QoL**
Symptoms (S)	-	0.67^†^	0.67^†^	0.56^†^	0.60^†^	0.53^†^	0.56^†^	0.39^†^	0.32	0.50^†^
Fatigue/Sleep (FT)		-	0.75^†^	0.75^†^	0.65^†^	0.54^†^	0.59^†^	0.36^†^	0.19	0.64^†^
Bother-Physical Health (B)			-	0.75^†^	0.79^†^	0.56^†^	0.79^†^	0.45^†^	0.08	0.78^†^
Activity (A)				-	0.63^†^	0.47^†^	0.62^†^	0.49^†^	-0.02	0.74^†^
Psychological (PH)					-	0.73^†^	0.73^†^	0.60^†^	-0.03	0.76^†^
Fear (FR)						-	0.58^†^	0.48^†^	0.07	0.58^†^
Social Activity (SA)							-	0.59^†^	0.04	0.70^†^
Work (W)								-	0.08	0.66^†^
Reproductive Health (RH)									-	0.13
Overall QoL (QoL)										-

^† ^p < 0.05

In addition to examining the correlations within the ITP-PAQ scales, construct validity was also assessed by comparing the ITP-PAQ scale scores with those of the CES-D and the SF-36. The CES-D was negatively correlated with all ITP-PAQ scales, except for the Reproductive Health scale. Other than the Reproductive Health scale, Pearson correlations ranged from -0.37 to -0.70 (p < 0.05) (data not shown). Most of the ITP-PAQ scales were moderately correlated with the SF-36 scales; however, the Reproductive Health scale was not significantly correlated with any of the SF-36 scales.

The mean SF-36 scores of the subjects with ITP were compared to those of the general U.S. population norms [15]. Results from t-tests indicate that there were statistically significant differences (p < 0.05) in SF-36 mean scores between subjects with ITP (range, 43.04–72.86) and the general U.S. population (range, 60.86–84.15). Subjects with ITP reported lower scores on each SF-36 scale compared to the US norm (data not shown).

### Known groups validity

**Figure 1:**
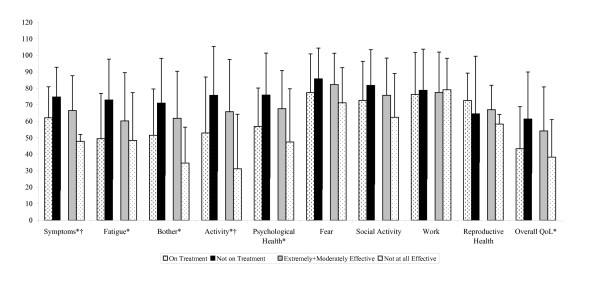
**Known groups validity: Comparison of scale scores by known groups**. *Statistically significant differences between subjects who were currently on treatment and subjects who were not on treatment (p < 0.01). ^†^Statistically significant differences between subjects who indicated that their treatment was extremely to moderately effective and subjects who indicated that their treatment was not effective at all (p < 0.05).

Subjects were categorized into two groups according to gender, splenectomy status, current ITP treatment status, subject's perception of the effectiveness of ITP treatment, and time elapsed since ITP diagnosis. When subjects were grouped according to gender or splenectomy status, no statistically significant differences were observed for any of the ITP-PAQ scales (data not shown). Subjects who were currently receiving treatment for ITP reported lower scores on all ITP-PAQ scales compared to subjects who were not currently receiving treatment. Statistically significant differences (p < 0.01) were reported for the following ITP-PAQ scales when subjects were categorized by treatment status: Symptoms, Fatigue/Sleep, Bother-Physical Health, Activity, Psychological Health, and Overall QoL (Figure 1). When subjects were categorized by effectiveness of ITP treatment, statistically significant differences (p < 0.05) were observed for the Symptoms and Activity scales (Figure 1), while statistically significant differences were only found for the Psychological Health scale when subjects were categorized according to time elapsed since ITP diagnosis (data not shown). Subjects who had been diagnosed with ITP for < 1 year had a lower mean score on the Psychological Health scale compared to subjects who had been diagnosed with ITP for at least one year (50.38 vs. 66.46, respectively; p = 0.02) (data not shown).

## Discussion

The goal of this study was to develop and undertake initial validation analyses of the ITP-PAQ as a tool for measuring HRQoL specifically related to adult subjects with ITP. The results of this study provide preliminary evidence of the reliability and validity of the ITP-PAQ in this population.

The results indicate that, with the exception of the Reproductive Health scale, the questionnaire has good internal consistency. The Reproductive Health scale may not have reached an acceptable level because the items could in fact be measuring slightly different concepts. For example, the Reproductive Health scale includes items that assess symptom bother related to menstruation in addition to items that ask how ITP impacts reproductive choices, such as becoming pregnant, giving birth, and adopting children. Perhaps, the symptom bother items in this scale may fit more appropriately with the Bother-Physical Health scale, and the reproductive choice items could comprise a separate scale.

Most of the ITP-PAQ scales also demonstrated acceptable test-retest reliability, even though the time interval between test and retest administrations of the questionnaire exceeded the targeted time interval of seven to ten days. However, two scales, the Bother-Physical Health and Activity scales, reported ICC values below the acceptable value of 0.70. In addition to the lag between the two administrations of the questionnaire, subjects may have experienced an increase in bother and/or a decrease in activity due to ITP during the extended time interval. Additionally, the comparatively low ICC values of the Bother-Physical Health and Activity scales may be due in part to the relatively low number of items contained in each of these scales (four and two items, respectively) compared to the Symptoms scale which contains six items.

In general, the construct validity of the questionnaire was supported by inter-scale correlations. As expected, the Bother-Physical Health, Symptoms, Fatigue/Sleep, and Activity scales were more strongly correlated to one another than with other scales. However, the Reproductive Health scale had a lower internal consistency reliability and it was weakly correlated with the ITP-PAQ scales, the SF-36, and the CES-D, possibly due to the differing concepts measured by the items within this scale or the all-female sample.

Most of the ITP-PAQ scales were moderately correlated with the SF-36 scales and the CES-D; however, correlations between some of the scales were < 0.40 (e.g., Fear and SF-36 Mental Health, 0.30; p < 0.05). This low correlation could be due to the ITP-PAQ assessing fear associated with ITP (e.g., fear of having a bleeding episode), while the SF-36 provides a more general assessment of mental health issues (e.g., felt downhearted and blue).

The known-groups validity results indicate that some of the ITP-PAQ scales (Symptoms, Fatigue/Sleep, Bother-Physical Health, Activity, Psychological Health, and Overall QoL scales) were able to differentiate ITP subjects who were currently receiving ITP treatment from those who were not receiving treatment for ITP, providing preliminary evidence of the ITP-PAQ's ability to distinguish between groups known to be different. However, the ITP-PAQ scales were generally unable to distinguish between subjects when they were grouped by gender, splenectomy status, perceived effectiveness of treatment and length of time since ITP diagnosis. The ITP-PAQ may not be able to differentiate between female and male subjects because the disorder may affect females and males similarly. Additionally, significant differences may not have been observed between subjects who have undergone splenectomy and subjects who have not because 55% of subjects who had a splenectomy indicated that it did not cure their ITP. Specifically, the known-groups could be defined as 'subjects without a splenectomy' versus 'subjects with a failed splenectomy' (for whom QoL likely worsened) versus 'subjects with a successful splenectomy' (for whom QoL may have improved). In the future, to assess whether the ITP-PAQ scales can differentiate between groups of subjects, it may be worthwhile to categorize subjects by a more clinically relevant measure, such as platelet count.

Several limitations should be considered when interpreting our findings. Subjects were drawn from a convenience sample. The study population was fairly homogeneous, comprised primarily of Caucasian female subjects. The data was validated using only patient-reported data collected via questionnaire. The lack of clinical data in this initial validation study will be addressed in on-going pivotal trials that will collect clinical data such as platelet counts and platelet response. In addition, the time interval between the initial and retest administration of the questionnaire may have been too lengthy to properly evaluate the test-retest reliability. Because 25% of subjects did not complete the questionnaire within the targeted fifteen day interval, those subjects may have undergone clinical changes that may have affected their responses. In future validation studies platelet counts or type of platelet response should be used to identify a stable cohort for the test-retest analyses. Furthermore, the criteria used to categorize the subjects for the known groups validity evaluation may not have been sufficient to allow for the ITP-PAQ scales to detect differences between groups. Grouping the subjects by a different criterion, such as a relevant clinical measure, may bolster the findings for its known groups validity.

## Conclusion

The primary goal of this manuscript was to describe the development of a new ITP-specific HRQoL questionnaire for adults with ITP and to present our initial findings on the psychometric properties of this questionnaire. The results of this initial validation study indicate that the questionnaire generally has acceptable reliability and validity. We plan to conduct additional analyses using more objective clinical measures such as platelet counts as a criterion for known groups validity. Further validation work should also be conducted to assess its responsiveness and to estimate its minimal clinical important difference value so that it can become a more widely used HRQoL measure in the ITP population.

## Competing interests

The validation study design, analysis, interpretation of results, and the writing of the manuscript represent the joint collaboration of all authors of this study, which was funded solely by Amgen, Inc, Thousand Oaks, California, USA. Ovation Research Group provided no additional funding for this study. The decision to submit this manuscript for publication was subject to the approval of Amgen, Inc. and all authors.

Gary Okano and Janet Nichol are employees of Amgen, Inc. James Bussel is an employee of Weill Cornell Medical Center. James George is employed by the University of Oklahoma Health Sciences Center. Robert McMillan is a Professor Emeritus of the Scripps Research Institute. Susan Mathias is an employee of Ovation Research Group.

## Authors' contributions

SDM supervised the interpretation of the results from the validation study, and drafted the manuscript. JBB, JNG, RM, and JLN provided clinical expertise in the development of the questionnaire, and participated in the design and execution of the study. GJO assisted in interpreting the results and drafting the manuscript. All authors read and approved the final manuscript.
